# Proteomic analysis revealed the potential usefulness of faecal extracellular vesicles in colorectal cancer diagnosis

**DOI:** 10.1038/s41598-026-35255-5

**Published:** 2026-01-08

**Authors:** Yusuke Murakami, Nozomi Sakamaki, Yoshiyuki Ohiro

**Affiliations:** Fundamental Research Laboratory, Research and Development Division, Eiken Chemical Co., Ltd., 143 Nogi, Nogi-machi, Shimotsuga-gun, Tochigi 329-0114 Japan

**Keywords:** Colon, Colorectal cancer, Extracellular vesicle, Proteomics, Faeces, Biochemistry, Biomarkers, Cancer, Gastroenterology

## Abstract

Colorectal cancer causes many deaths annually worldwide. We aimed to develop a new diagnostic method for colorectal cancer by analysing the protein profiles of extracellular vesicles in faeces. Enrichment analysis of proteomic data on extracellular vesicles highly purified from faeces by density gradient centrifugation revealed that proteins detected only in extracellular vesicles were significantly enriched in proteins derived from the colon. Notably, proteins detected only in faecal suspensions showed no enrichment in colon-derived proteins, suggesting that faecal extracellular vesicles can be used to detect changes in the protein profile that occur in the colon. In addition, we identified four candidate biomarkers among the proteins that were altered in the faecal extracellular vesicles of patients with colorectal cancer identified by proteomic analysis. By comparing different healthy control samples, olfactomedin-4 (OLFM4) and galectin-3-binding protein (LGALS3BP) in faecal extracellular vesicles were significantly increased in patients with colorectal cancer. This study shows that faecal extracellular vesicles are a promising biomarker resource for colorectal diseases, including colorectal cancers. Further analysis using faecal extracellular vesicles may lead to the development of novel diagnostic methods for colorectal cancer.

**Supplementary Information:**

The online version contains supplementary material available at 10.1038/s41598-026-35255-5.

## Introduction

Colorectal cancer (CRC) is the third most common cancer in terms of incidence and second most common cause of mortality, causing 900,000 deaths annually worldwide. It is the leading cause of cancer-related deaths worldwide and accounts for 10% of the total cancer burden^1^. Recently, the incidence of CRC has increased worldwide. Although screening programs are available in many countries, CRC is often detected at an advanced stage. CRC progresses slowly, and can be cured if detected early and treated appropriately^2^. Colonoscopy is the most commonly used and accurate diagnostic method. However, this procedure is highly technical and invasive. One of the typical symptoms of CRC is the presence of blood in the faeces, called occult blood. This characteristic is utilised in faecal immunochemical testing (FIT), which is widely used as a screening method for CRC. In many clinical trials, FIT was useful for reducing CRC mortality, and is less costly and more effective than other screening methods^3,4^. However, FIT is not suitable for detecting advanced adenomas and early cancers because patients with these diseases do not have occult blood in their faeces. Therefore, there is a need to identify new biomarkers for advanced adenoma and early-stage CRC^5^.

Recently, many studies have confirmed that circulating tumour, cell-free, and methylated DNA may be prominent biomarkers for CRC. Epi proColon, which detects methylated septin9 in blood cfDNA, has been approved by the Food and Drug Administration (FDA) as a blood-based screening test. However, the Preventive Services Task Force has not approved its use for CRC^6–8^. In terms of faeces-based testing, the multi-targeted stool DNA test is used as a non-invasive FDA-approved screening method for CRC. ColoGuard is a stool DNA-based CRC screening method that combines DNA markers and FIT with a sensitivity and specificity of 93.9% and 90.9%, respectively^9,10^. ColoSense is a stool RNA-based CRC screening method developed by Geneoscopy that uses mRNA markers, FIT, and patient information, such as smoking status. It has a sensitivity of 94% for CRC and 46% for advanced adenomas, and has recently obtained FDA approval^11^. Recent studies have explored miRNA-based tests^11^ using blood^12^ and faecal samples^13^, demonstrating a higher sensitivity than that of FIT, but have not yet been commercialised. Although, a variety of tests have been developed, most of them are more expensive and cumbersome than FIT and have lower specificity. Therefore, there is a need to develop new tests to complement existing colorectal cancer screening methods.

Extracellular vesicles are lipid bilayer membrane-enclosed particles released from cells that encapsulate various molecules, such as proteins and microRNAs, and play important roles in intercellular signalling^14–16^. Extracellular vesicles are present in large numbers in bodily fluids, such as blood and urine. Many researchers have focused on them as a new source for liquid biopsies^17,18^. Recently, host-derived extracellular vesicles have been reported to be present in faeces^19,20^. Therefore, we believe that our research should focus on faecal extracellular vesicles (fEVs). Although there are many studies on biomarkers using extracellular vesicles in CRC, most of these studies have been performed on blood samples^21–24^. Extracellular vesicles are released directly from tumour cells, therefore, the detection of tumour-released extracellular vesicles in the blood may lead to the development of a useful diagnosis. However, given the progression of CRC, tumour-derived components may be released into the intestinal tract earlier than they entering the blood^25^. That is, luminal detachment occurs earlier in the process of colorectal tumourigenesis than vascular invasion, therefore, targeting fEVs for biomarker discovery may be effective than blood analysis for earlier cancer detection Many researchers have performed proteomic analyses of faecal samples to identify new biomarkers for CRC^26–28^. However, few studies have analysed fEVs derived from the host rather than from intestinal bacteria. In addition, whether fEVs are truly useful for diagnosis has not been analytically confirmed. Based on this perspective, we evaluated the potential utility of fEVs for colorectal cancer diagnosis by purifying fEVs and analysing their protein profiles.

## Result

### Purification of fEVs

**Fig. 1:**
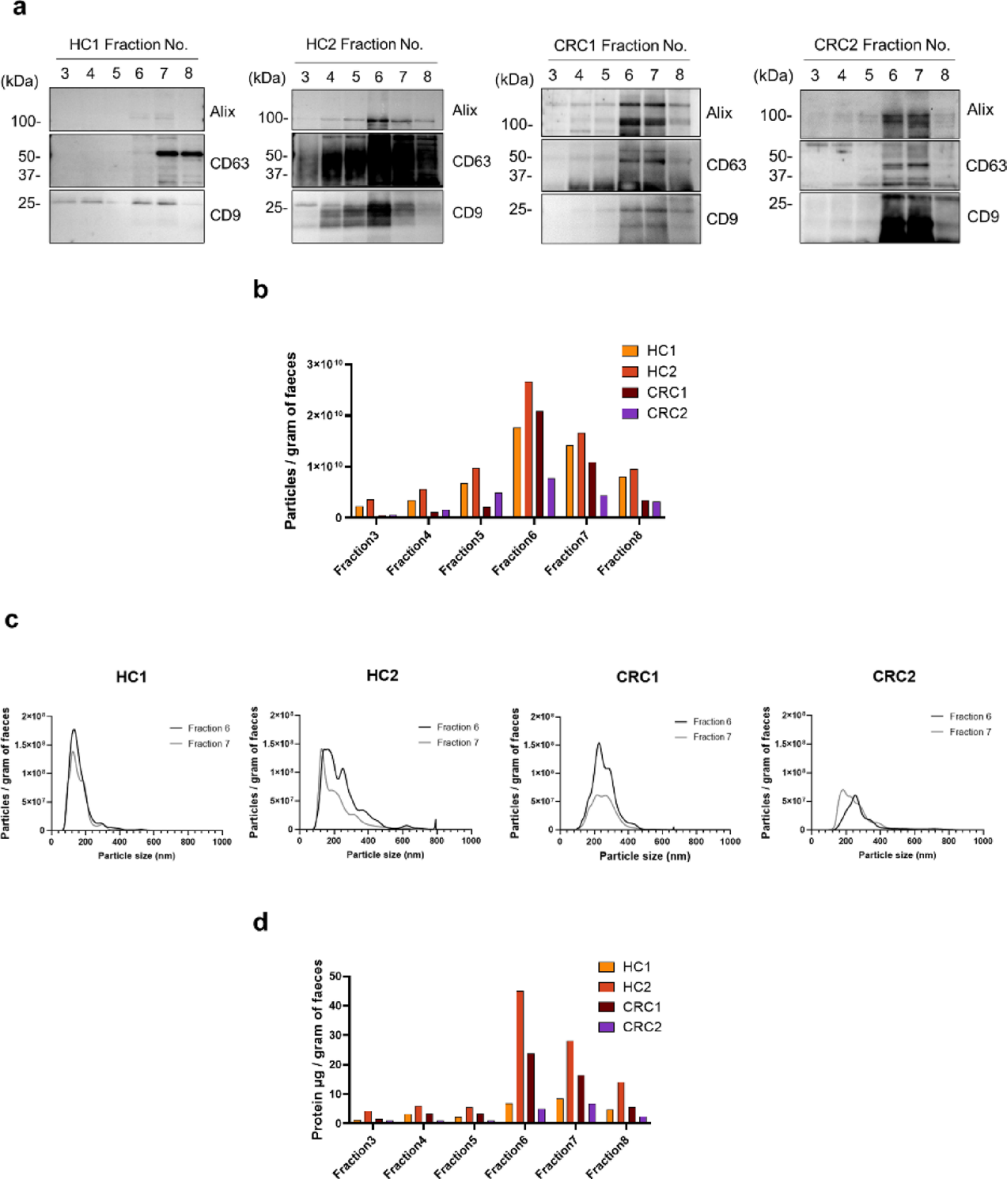
Analysis of faeces fractionated using density gradient centrifugation. Faecal fractions from HCs and patients with CRC. (**a**) The levels of Alix, CD63, and CD9 proteins in the fractions were confirmed using western blotting. (**b**) Particles per gram of faeces in the fraction obtained using density gradient fractionation of faeces analysed using NanoSight. (**c**) Particle size distributions in fractions 6 and 7. (**d**) Protein concentration per gram of faeces in the fraction obtained using density gradient fractionation. HC, healthy control; CRC, colorectal cancer.

To analyse extracellular vesicles in faeces, we purified fEVs from the faeces of two healthy control (HC) individuals and patients with CRC using a purification method combining multiple centrifugation and density gradient fractionation. To confirm which fraction of the density gradient fractions contained extracellular vesicles, we analysed extracellular vesicle protein markers using western blotting (Fig. 1a and Supplementary Fig. S1). Alix, CD63, and CD9 were detected in fractions 6 and 7. The densities of fractions 6 and 7 were 1.107–1.129 g/mL, which were close to the values reported in previous studies for fractions yielding extracellular vesicles^29^. To confirm the distribution of extracellular vesicle particles in each fraction, particle analysis of extracellular vesicles was performed using NanoSight. According to the results of particle distribution measurements, most particles were observed in fractions 6 and 7 (Fig. 1b). Histograms of particle counts and sizes indicated that fractions 6 and 7 contained particles with diameters of 100–300 nm (Fig. 1c). Finally, measurement of the protein concentration in each fraction revealed that fractions 6 and 7 contained the most protein (Fig. 1d). These results suggested that most of the extracellular vesicles purified from faeces by density gradient centrifugation were present in fractions 6 and 7. Therefore, we decided to use a mixed sample of these two fractions as the purified fEVs.

### Proteomic analysis of fEVs

**Fig. 2:**
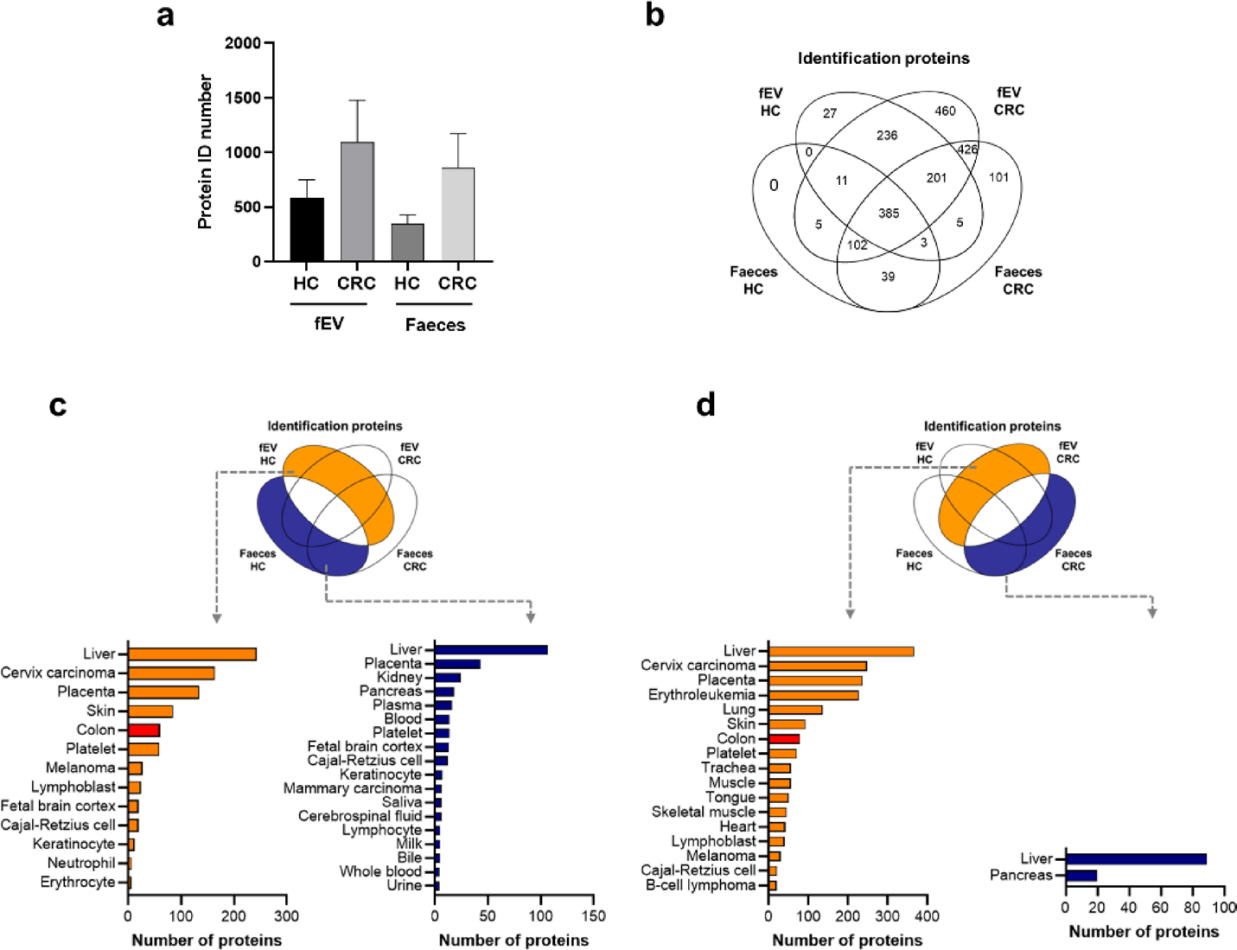
Analysis of fEV proteins identified using proteomic analysis. Results of identified proteins obtained using proteomic analysis of fEVs and faecal suspensions from four HC and four CRC samples (**a**) Number of proteins identified using proteomic analysis. (**b**) Venn diagram comparing the proteins identified using proteomic analysis in each sample. (**c**) Enrichment analysis results for a group of proteins identified only in fEVs (left) or faecal suspensions (right) in HC samples. Red bars indicate colon-related tissues. (**d**) Enrichment analysis results for a group of proteins identified only in fEVs (left) or faecal suspensions (right) of a CRC sample. Red bars indicate colon-related tissues. HC, healthy control; CRC, colorectal cancer; fEV, faecal extracellular vesicle.

Extracellular vesicle purification from faeces using density gradient centrifugation was successful, therefore, we performed a quantitative proteomic analysis to characterise the purified extracellular vesicles. Purified fEVs and faecal suspensions from each sample were subjected to proteomic analysis, except for one patient with CRC who lacked a faecal sample. After obtaining trypsin-digested peptides from the purified extracellular vesicles and faecal suspensions, Data independent acquisition (DIA) proteomic analysis was performed using mass spectrometry (MS). Consequently, 2001 proteins were identified. The number of proteins identified in each sample is shown in Fig. 2a and b, and Supplementary Table S1. To evaluate whether the purification and concentration procedures for fEVs were successful for proteomic analysis, we confirmed the quantitative levels of extracellular-vesicle–associated protein markers in all samples^30^. As expected, fEVs exhibited higher levels of these markers compared with faecal suspensions (Supplementary Fig. S2). More proteins were identified in both extracellular vesicles and faecal suspensions in samples from patients with CRC than those in samples from HCs. In addition, more proteins were identified in extracellular vesicles than those in faecal suspensions from both HCs and patients with CRC. The distribution of identified proteins is shown in a Venn diagram (Fig. 2b). In total, 385 proteins were identified as common to all samples. The proteins identified only in extracellular vesicles from HCs, patients with CRC, and faecal suspensions from patients with CRC were 27, 460, and 101, respectively. No proteins were identified in the faecal suspensions of HCs. Overall, several proteins in faecal suspensions and extracellular vesicles were different. A difference was also found between HCs and patients with CRC.

### Enrichment analysis of proteins identified using proteomic analysis of fEVs and faecal suspensions

We performed an enrichment analysis to characterise the differences in the protein profiles of fEVs and faecal suspensions. Enrichment analysis of protein groups identified only in extracellular vesicles and faecal suspensions was performed using data from HCs and patients with CRC patients to determine the differences in the tissues from which the protein groups were derived. In total, 469 proteins were identified only in extracellular vesicles and 146 proteins were identified only in faecal suspensions. Enrichment analysis of these protein groups showed statistical enrichment of proteins derived from 13 to 18 different tissues, respectively (*p* < 0.05, FDR < 5%; Fig. 2c). Notably, the enrichment of proteins derived from the lower gastrointestinal tissue (colorectal) was only observed in the group of proteins identified in extracellular vesicles. In patients with CRC, 712 and 148 proteins were detected in extracellular vesicles and faecal suspensions, respectively. Enrichment analysis of these protein groups in patients with CRC also showed enrichment from 17 different tissues for the protein groups identified only in fEVs and two different tissues for the protein groups identified only in the faecal suspension (*p* < 0.05, FDR < 5%, Fig. 2d). In patients with CRC with the same pattern as that in HCs, enrichment of proteins derived from colon tissue was observed only in the group of proteins identified in fEVs. When the same analysis was performed on individual samples from four pairs of HCs and three pairs of patients with CRC, enrichment of proteins derived from colon tissue occurred only in the protein group identified in extracellular vesicles in all samples, whereas no enrichment of proteins derived from colon tissue was observed in the protein group identified only in the faecal suspension (Supplementary Fig. 3, 4). The results of the enrichment analysis indicated that fEVs contained colon tissue proteins that were common between HCs and patients with CRC. These findings demonstrate that the protein composition of fEVs is distinct from that of faecal suspensions and that fEVs contain higher levels of proteins originating from colon tissue.

### Evaluation of the usefulness of fEVs in the diagnosis of CRC

**Fig. 3:**
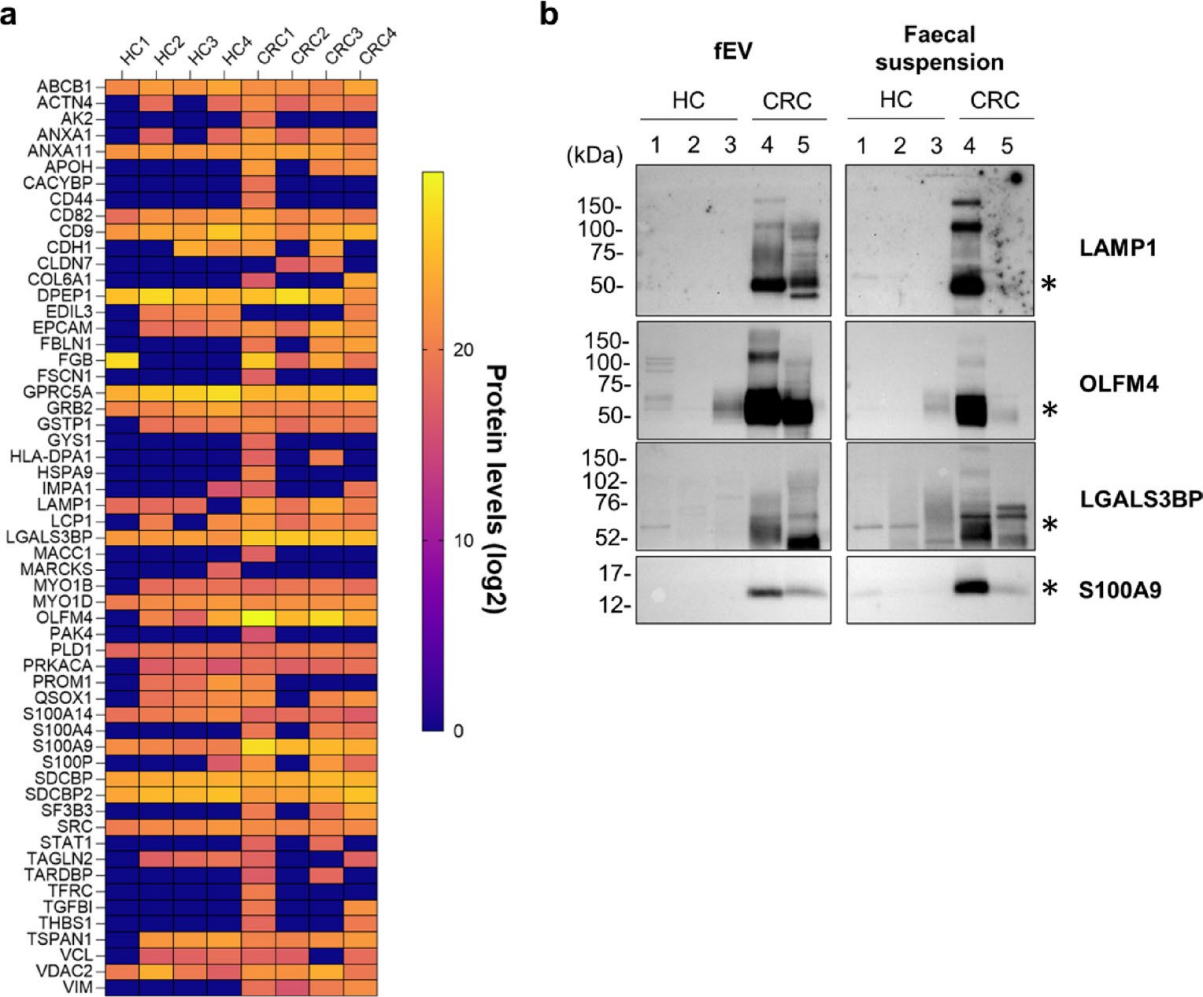
Evaluation of candidate biomarkers found in proteomic analysis of fEVs. (**a**) Heat map showing proteomic analysis quantitative values of 57 candidate biomarker proteins for CRC in fEVs selected from published articles. (**b**) Western blotting comparison of protein levels in fEVs and faecal suspensions of HC and CRC groups for candidate biomarker proteins, LAMP1, OLFM4, LGALS3BP, and S100A9. *The indicated band size predicted from the amino acid sequence. HC, healthy control; CRC, colorectal cancer; fEV, faecal extracellular vesicle.

To confirm the potential utility of fEV-derived proteins for CRC diagnosis, we explored candidate biomarkers among the proteins identified by proteomic analysis. We used Embase, a comprehensive medical database for biomedical research, to identify proteins that showed quantitative changes in extracellular vesicles released from CRC cells and tissues. In total, 155 proteins were identified. Of these, 57 were detected in the proteomic analysis of fEVs. Furthermore, of these 57 proteins, 42 showed more than 2-fold or less than 0.5-fold change between HCs and patients with CRC (Fig. 3a). To validate the changes in these proteins, we performed western blotting with specific antibodies. Four proteins, olfactomedin-4 (OLFM4), lysosome-associated membrane glycoprotein 1 (LAMP1), galectin-3-binding protein (LGALS3BP, and S100A9, were selected as candidate proteins based on their alterations and the quantitative value of the proteomic analysis. Western blot analysis was performed using 2.5 µg of protein extracted from fEVs and faecal suspensions obtained from three healthy controls (HCs) and two patients with colorectal cancer (CRC). In faecal suspensions, OLFM4, LAMP1, and S100A9 were detected in only one of the two CRC samples, whereas LGALS3BP was detected in both samples (Fig. 3b and Supplementary Fig. S5).

A comparison between the quantitative values obtained from Western blot analysis and those from proteomic analysis demonstrated overall concordance across samples (Supplementary Table S2).

In the fEVs, strong bands were detected for all candidate proteins in both CRC samples. In HCs, various bands were detected in faecal suspensions, especially for LGALS3BP, whereas few bands were detected in the extracellular vesicles. Furthermore, various band patterns were observed for LAMP1, OLFM4 and LGALS3BP. As all three proteins are potentially glycosylated, they are considered to be glycoprotein isoforms^31–35^. These results suggest that we could obtain several candidate proteins as CRC biomarkers from fEV proteomic analysis data. Thus, fEVs may be useful in the diagnosis of CRC.

### Validation of candidate proteins for CRC biomarkers obtained from fEVs

**Fig. 4:**
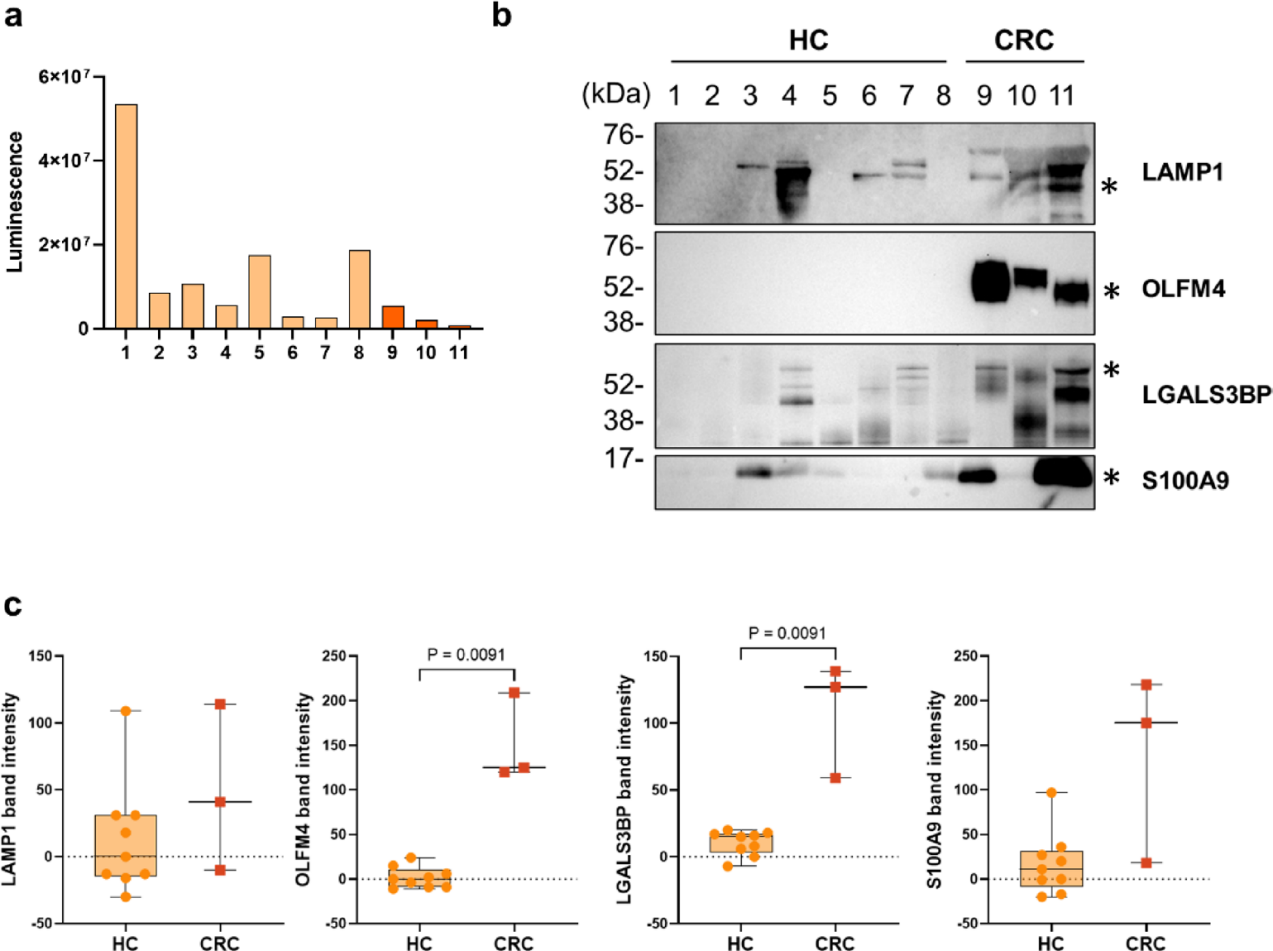
Validation of candidate biomarker proteins using different HC groups. (**a**) Measurement of extracellular vesicle levels in faecal suspensions from eight HCs, different from the samples used for proteomic analysis, and three faeces samples from patients with CRC, same as samples used for proteome analysis, using a sandwich assay system with a monoclonal antibody against CD63. (**b**) Comparison of the levels of candidate biomarker proteins, LAMP1, OLFM4, LGALS3BP, and S100A9, in fEVs from HC and CRC groups using western blotting. *The indicated band size predicted from the amino acid sequence. (**c**) Western blot band intensity in (**b**) was quantified and represented in box-and-whisker plots. HC, healthy control; CRC, colorectal cancer; fEV, faecal extracellular vesicle.

To further investigate the diagnostic performance of the candidate biomarker proteins in patients with CRC, we validated these proteins in eight HC faecal samples that were different from those used in the proteomic analysis. The faeces samples of patients with CRC were the same as those used in the proteomic analysis. The fEVs were crudely purified using ultracentrifugation only, which is different from the purification method used for proteomic analysis. To avoid the effect of proteins derived from the faecal suspension contaminated by ultracentrifugation, we normalised the sample volume according to the levels of extracellular vesicles in the purified fEVs. CD63 is widely distributed in extracellular vesicles and is well known as a common extracellular vesicle marker; therefore, we normalised using CD63 levels in accordance with the method previously reported by Yoshioka et al.^36^. To assess the validity of normalization using CD63, we examined the relationship between CD63 quantification values obtained from the proteomic analysis and the particle counts of the analyzed samples (Supplementary Fig. S7). The results demonstrated a significant inverse correlation between CD63 levels and particle counts. The levels of CD63-positive extracellular vesicles in purified fEVs were measured using a sandwich assay system with a CD63 monoclonal antibody. The results are shown in Fig. 4a. Western blotting was performed using samples adjusted to equalise the levels of extracellular vesicles between samples based on the results of the CD63-positive extracellular vesicle measurement. All candidate biomarker proteins showed stronger bands in patients with CRC compared with those of HCs (Fig. 4b and Supplementary Fig. S8). The plots of quantified band intensities showed significant differences in OLFM4 and LGALS3BP between HCs and patients with CRC (U test, *p* = 0.0091 and *p* = 0.0091, respectively). These fEV proteins were suggested as potential CRC biomarkers (Fig. 4c).

## Discussion

In this study, fEVs were enriched with proteins derived from the colon and the advantages of using fEVs for the diagnosis of CRC was demonstrated. Moreover, four candidate biomarker proteins, which were found to be increased in the fEVs of CRC patients, were identified using proteomic analysis.

The proteomic analysis of faeces still faces many challenges because faeces are unique samples, such as differences in the properties of each sample and contamination. Therefore, it has not yet led to the discovery of useful biomarkers. Purification of fEVs can remove the complex matrix of faeces during the isolation of extracellular vesicles. In our proteomic analysis, we detected more proteins in fEVs than those in faecal suspensions. Targeting fEVs has advantages not only in early detection but also in providing more comprehensive proteomic analysis.

Enrichment analysis of proteins in fEVs showed that the fEVs were significantly enriched in proteins derived from the colon. In contrast, proteins derived from the colon were not enriched in the faecal suspensions. This observation was commonly observed in all seven samples analysed in the present study, HCs and patients with CRC. According to these results, it can be presumed that more molecules derived from the colon can be detected with higher sensitivity using fEV samples than those with faecal suspensions. Therefore, fEVs are useful for detecting changes originating from the colon and diagnosing colorectal diseases, such as CRC.

In biomarker discovery studies targeting extracellular vesicles, serum and plasma are often used as samples for CRC diagnosis^21–23^. However, extracellular vesicles derived from cells throughout the body circulate in the blood. Accordingly, information on extracellular vesicles derived from specific tissues and cells is limited^37,38^. This perspective also suggests that faeces, which are body fluids in direct contact with the tumour lesion, might be a useful diagnostic target. Zhang et al. reported fEVs as a useful CRC biomarker in 2023. They compared fEVs with CRC tissue and showed that proteins highly expressed in CRC tissue were also abundant in fEVs, and established biomarkers for fEVs^39^. However, protein expression patterns in extracellular vesicles and derived tissues do not completely match^40^. Therefore, we selected a list of proteins that fluctuate in extracellular vesicles derived from CRC, rather than proteins highly expressed in CRC cells or tissues, to search for candidate biomarkers. Our selected approach confirmed that 42 of the listed proteins, which comprised more than 70% of the 57 proteins identified in the proteomic analysis of fEVs, showed significant changes of 2-fold or more than 0.5-fold between HCs and patients with CRC. Among these, we established new fEV biomarkers, LAMP1, OLFM4, LGALS3BP, and S100A9. Particularly, OLFM4 and LGALS3BP were significantly increased in fEVs from patients with CRC.

However, the fEV biomarkers identified in this study were selected solely by comparing quantitative values among a limited number of samples and were not chosen based on associations with patients’ clinical characteristics (e.g., tumour size or stage). This study highlights the advantage of using fEVs rather than faecal suspensions for biomarker development in colorectal cancer. Therefore, proteomic analyses of fEVs using a larger cohort with well-defined clinical information may lead to the identification of biomarkers with potential clinical applicability. This study had several limitations. First, it was difficult to obtain faecal samples with detailed diagnostic information; therefore, the clinical background of each patient (e.g. stage, presence or absence of metastases, and medication information) was unknown. Therefore, it was not possible to determine how the biomarkers identified in this study varied with the progression and metastatic nature of CRC. Although previous studies, particularly on OLFM4, have reported increased protein levels in colorectal adenomas and early-stage colorectal tumours^34^, whether the biomarkers identified in this study are actually increased in the fEVs of patients with early-stage CRC needs to be confirmed in further studies. Second, the number of clinical samples used in this study was insufficient. In the present study, only four cases of faeces from patients with CRC were available; therefore, the candidate biomarker proteins obtained from the search could not be evaluated in different groups of patients with CRC. However, the performance of the four candidate biomarker proteins was confirmed in a HC group using eight different cases from the discovery. Furthermore, considerable variability among individual clinical samples was also observed in fEVs, as indicated by differences in the Western blot band patterns. These differences are likely attributable to variations in the extent of N-linked glycosylation of these proteins, as well as differences in the degree of proteolytic degradation, which are commonly observed in stool-derived extracellular vesicle proteins. Therefore, analyses using a larger number of samples will be required to obtain results that smooth out inter-sample variability. In addition, this study could not clearly distinguish EVs from other cellular debris in all samples. Consequently, the results should be interpreted with caution, taking into account the potential influence of such contamination.

In conclusion, we demonstrated the usefulness of fEVs in the diagnosis of CRC by performing a high-sensitivity proteomic analysis and proteins contained in fEVs could be potential biomarkers for colorectal cancer. In the future, fEVs proteomic analysis using a large number of samples with available clinical backgrounds could lead to the discovery of new biomarkers for colorectal cancer.

## Methods

### Faecal sample collection

The protocol for this study was conducted in accordance with the Declaration of Helsinki, and informed consent was received from all individuals and patients participating in this study. The study protocol was approved by the Ethics Review Board of Eiken Chemical Co., Ltd. (ECC) (Tochigi, Japan) (86 − 006, 86 − 002, 87 − 007). Collection of faeces from HC individuals was performed during health examinations at ECC. HC inclusive criteria were: ≥ 56 years of age and faecal occult blood test result below the reference value. The faeces were collected in containers and submitted by the HC individuals, aliquoted into the required volume, and stored at -80 °C until analysis. Faecal material from patients with CRC was purchased from Trina Bioreactives AG (Naenikon, Switzerland).

### Purification of fEVs for proteomic analysis using density gradient fractionation

Purification of fEVs was performed by using a modified version of the method described by Yang et al.^41^. Frozen faeces (2–7 g) were suspended in 50 mL of phosphate-buffered saline (PBS) with a protease inhibitor cocktail (Roche Diagnostics, Mannheim, Germany) and placed on ice for 30 min. After resuspension, the suspensions were centrifuged at 3,000 × g for 30 min, the resulting supernatants were filtered through a filter paper (Advantech, Tokyo, Japan) and the filtrates were centrifuged at 40,000 × g for 90 min at 4 °C in a SW 28 swinging-bucket rotor (15,000 rpm) (Beckman Coulter, Brea, CA, USA). The faecal suspensions used for proteomic analysis were subjected centrifugation at 40,000 × g. For extracellular vesicle purification from faeces, the supernatants were filtered through a 0.45-µm filter (Whatman, Kent, UK) after centrifugation. Then the filtrates were centrifuged at 141,000 × g for 150 min at 4 °C in a SW 28 swinging-bucket rotor (28,000 rpm) (Beckman Coulter) to obtain a crude purification of fEVs as precipitate. Next, OptiPrep (Axis-Shield, Oslo, Norway) density gradient centrifugation was performed for crude purification of fEVs^42^. OptiPrep (60% w/v) and buffer1 (0.75 M sucrose/10 mM Tris, pH 7.4) were mixed to prepare a 40% iodixanol solution. Subsequently, the 40% iodixanol solution was mixed with buffer 2 (0.25 M sucrose/10 mM Tris, pH 7.4) to prepare 20%, 10%, and 5% iodixanol solutions respectively. The 3 mL of the 40% iodixanol solution was transferred to a ultracentrifuge tube (13.2 mL Open-Top Thinwall Ultra-Clear Tube, Beckman Coulter) bottom, followed by loading with a 3 mL 20%, 3 mL 10%, and 2.5 mL 5% iodixanol gradient, and the top layer was overlaid with 0.5mL of crude purified fEVs, followed by centrifugation at 100,000 × g for 18 h at 4 °C in a SW 41 Ti swinging-bucket rotor (25,000 rpm) (Beckman Coulter). After centrifugation, 1-mL fractions were collected from the upper layer and divided into fractions 1–12. Then, 11 mL of PBS was added to each fraction, followed by centrifugation at 141,000 × g for 120 min, and the precipitates were suspended in 100 µL of PBS. The ultracentrifuge was operated using OptimaTM L-90 K ultracentrifuge (Beckman Coulter).

### Characterisation of fEVs

The particle size and number of extracellular vesicles purified from faeces were analysed using a NanoSight NS300 (Malvern Panalytical, Malvern, UK) based on nanoparticle tracking analysis. The particle size and concentration were measured using extracellular vesicles diluted 100–1,000-fold in PBS filtered through a 0.22-µm filter as the sample. The total protein levels of the purified extracellular vesicle and faecal suspension were determined using the BCA protein assay (Thermo Fisher Scientific, Waltham, MA, USA) for protein concentration after the samples were diluted at least 10-fold with PBS.

### Proteomic analysis of purified fEVs and faecal suspensions

Proteomic analysis of fEVs and faecal suspensions was conducted at the Kazusa DNA Research Institute (Chiba, Japan) using a Q-Exactive HF-X (Thermo Fisher Scientific) via the DIA proteomic analysis service (Promega, Madison, WI, USA). Briefly, fEVs (200 ng) and faecal suspensions were digested overnight at 37 °C by adding 500 ng of Tripsin/Lys-C Mix (Promega) after reduction-alkylation. After digestion, the samples were desalted using a reversed-phase spin column (GL Sciences, Tokyo, Japan) and analysed. The sample (200 ng) was separated using an UltiMate 3000 RSLC nano LC System (Thermo Fisher Scientific), followed by MS/MS analysis (DIA MS). The obtained data were analysed using DIA-NN^43^. Only proteins with precursor and protein FDR < 1% were identified and quantified. Quantitative values were calculated as relative quantitative values using DIA-NN.

### Western blotting

For detection of protein markers of extracellular vesicles, 17.5 µL of each fraction containing extracellular vesicles purified using density gradient centrifugation was used (Fig. 1a). To detect candidate biomarker proteins, 2.5 µg of extracellular vesicles (mixture of fractions 6 and 7) purified using density gradient centrifugation was used (Fig. 3b). For candidate biomarker proteins in extracellular vesicles purified by ultracentrifugation, the sample volume was adjusted to equalise the signal of the measurement system to CD63-positive extracellular vesicles (Fig. 4b). In all cases, the prepared samples were loaded onto a 10–20% gradient gel (ATTO, Tokyo, Japan), subjected to SDS-PAGE, and transferred to a PVDF membrane. Protein-transferred membranes were blocked with PVDF Blocking Reagent for Can Get Signal (TOYOBO, Osaka, Japan) and then reacted with one of the following primary antibodies: anti-human Alix Rabbit polyclonal (Proteintech, Rosemont, IL, USA), anti-human CD63 Rabbit polyclonal (Proteintech), anti-human CD9 Rabbit polyclonal (Proteintech), anti-human LAMP1 Rabbit monoclonal (Cell Signalling Technology, Danvers, MA, USA), anti-human OLFM4 Rabbit monoclonal (Cell Signalling Technology), anti-human LGALS3BP Rabbit polyclonal (Proteintech), anti-human S100A9 Rabbit monoclonal (Cell Signalling Technology) antibody. Details of the antibodies used in this study are provided in the Supplementary Table S3. The primary antibody was diluted using Can Get Signal Immunoreaction Enhancer Solution (TOYOBO) and incubated overnight at 4 °C. After washing, the membrane was incubated with a secondary antibody, horseradish peroxidase-labelled anti-rabbit immunoglobulin G (Cell Signalling Technology). Protein bands were detected using an ECL Prime kit (GE Healthcare, Piscataway, NJ, USA) and visualised using an iBright Imaging System (Thermo Fisher Scientific). Protein bands were quantified using ImageJ software v1.54d (Rasband, W.S., NIH, Bethesda, MD, USA).

### Measurement of CD63-positive extracellular vesicle levels

The BLEIA method^44,45^ based on bioluminescence was used to detect CD63-positive fEVs using a fully automated analyser, BLEIA-1200 (Eiken Chemical, Tokyo, Japan). The fEVs purified from faeces were used as samples. As the first step of the reaction, 50 µL of the sample was mixed with 50 µL of magnetic particles coated with anti-human CD63 monoclonal antibody (SHI-EXO-M01, Cosmobio, Tokyo, Japan), and biotinylated anti-human CD63 monoclonal antibody (SHI-EXO-M 02-B, Cosmobio) were mixed and reacted at 37 °C for 15 min. Then, washing with BLEIA washing solution (ECC) was performed, and 80 µL of Tris buffer containing streptavidin and biotinylated luciferase was added, followed by a secondary reaction at 37 °C for another 15 min. After the secondary reaction, washing with BLEIA washing solution (ECC) was performed and 100 µL of luminescent substrate solution (ECC) was added to detect the luminescence signal.

### Statistical analysis

Statistical analysis was conducted using BellCurve for Excel (Social Survey Research Information, Tokyo, Japan) and GraphPad Prism software (GraphPad Software Inc., San Diego CA, USA). The Mann–Whitney U test was used to compare the two groups and results with *p* < 0.05 were considered statistically significant. Enrichment analysis of the protein groups differentially expressed in faecal extracellular vesicles and faecal suspensions was performed using the Database for Annotation, Visualisation, and Integrated Discovery (http://david.abcc.ncifcrf.gov/) (DAVID 2021) to analyse tissue-specific protein enrichment.

## Supplementary Information

Below is the link to the electronic supplementary material.

Supplementary Material 1

Supplementary Material 2

## Data Availability

The data for the proteomic analysis of faecal extracellular vesicles and faecal suspensions are presented in Supplementary Table S1.
