# Supplementary material for: Proteomic analysis revealed the potential usefulness of faecal extracellular vesicles in colorectal cancer diagnosis
Source: Sci Rep. 2026 Jan 8;16:4863. doi: 10.1038/s41598-026-35255-5 (PMC12873230; doi:10.1038/s41598-026-35255-5)
Supplement: Supplementary file 2 — Supplementary Material 2 [file 41598_2026_35255_MOESM2_ESM.pdf]

# Supplementary Information

## Title : Proteomic analysis revealed the potential usefulness of fecal extracellular vesicles in colorectal cancer diagnosis

Authors : Yusuke Murakami, Nozomi Sakamaki, Yoshiyuki Ohiro

## List of contents


- **Supplementary Table S1.** List of proteins identified in fEV. (This file is available separately as an Excel file.)
- **Supplementary Table S2.** Quantitative value of Western blotting in Fig 3b and proteome analysis of CRC biomarkers in fEVs and faecal suspensions.
- **Supplementary Table S3.** Antibodies used in Western blotting.
- **Supplementary Figure S1.** Whole images of western blotting analysis in Fig 1a.
- **Supplementary Figure S2.** Protein levels of extracellular vesicle markers in fEVs and faeces samples.
- **Supplementary Figure S3.** Enrichment analysis of individual HCs.
- **Supplementary Figure S4.** Enrichment analysis of individual patients with CRC.
- **Supplementary Figure S5.** Whole images of western blotting analysis in Fig 3b.
- **Supplementary Figure S6.** Primary antibody omission plot for western blotting analysis in Fig 3b.
- **Supplementary Figure S7.** Correlation between the quantification values of CD63 and the EV particles.
- **Supplementary Figure S8.** Whole images of western blotting analysis in Fig 4b.

**Supplementary Table S2. Quantitative value of Western blotting in Fig 3b and proteome analysis of CRC biomarkers in fEVs and faecal suspensions.**

| Group | Sample name | | | LAMP1 | | OLFM4 | | LGALS3BP | | S100A9 | |
|---|---|---|---|---|---|---|---|---|---|---|---|
| | Proteome | Fig 3b | Fig 4c | WB | Proteome | WB | Proteome | WB | Proteome | WB | Proteome |
| **fEVs** | HC1 | #1 | - | -1 | 2.8E+05 | 9 | 1.5E+06 | 2 | 4.8E+06 | 9 | 1.7E+06 |
| | HC2 | #2 | - | -8 | 3.2E+05 | -6 | 1.9E+05 | -6 | 7.5E+06 | 4 | 8.6E+05 |
| | HC3 | #3 | - | -10 | 0.0E+00 | 27 | 1.3E+07 | -5 | 3.2E+06 | 0 | 1.1E+06 |
| | CRC1 | #4 | #9 | 134 | 9.3E+06 | 153 | 6.7E+08 | 64 | 6.9E+07 | 45 | 1.7E+08 |
| | CRC2 | #5 | #11 | 94 | 1.1E+07 | 117 | 1.5E+08 | 68 | 3.9E+07 | 14 | 2.9E+07 |
| **faecal suspensions** | HC1 | #1 | - | -11 | 4.7E+06 | -12 | 1.5E+06 | -1 | 5.0E+06 | 1 | 4.6E+07 |
| | HC2 | #2 | - | -12 | 2.5E+06 | -9 | 5.1E+06 | 16 | 2.1E+07 | 3 | 6.0E+06 |
| | HC3 | #3 | - | -7 | 0.0E+00 | 13 | 6.0E+06 | 49 | 4.6E+07 | 10 | 5.0E+07 |
| | CRC1 | #4 | - | 165 | 1.3E+06 | 129 | 8.2E+07 | 127 | 9.5E+06 | 119 | 5.0E+08 |
| | CRC2 | #5 | - | 47* | 7.2E+06 | 21 | 4.9E+07 | 82 | 8.8E+06 | 41 | 5.2E+07 |

*The indicated values may not be accurate due to image distortion during quantification.
WB, Western blotting; HC, healthy control; CRC, colorectal cancer; fEV, faecal extracellular vesicle.

**Supplementary Table S3. Antibodies used in Western blotting.**

| Antigen | Clone / ID | Host | Manufacturer (Catalog number) | Dilution |
|---|---|---|---|---|
| OLFM4 | D1E4M XP | Rabbit | Cell Signaling (#14369) | 1:1,000 (WB) |
| LGALS3BP | Polyclonal | Rabbit | Proteintech (#10281-1-AP) | 1:500 (WB) |
| LAMP1 | D2D11 XP | Rabbit | Cell Signaling (#9091) | 1:1,000 (WB) |
| S100A9 | D5O6O | Rabbit | Cell Signaling (#72590) | 1:500 (WB) |
| CD63 | - | Rabbit | Proteintech (#25682-1-AP) | 1:1000 (WB) |
| CD9 | - | Rabbit | Proteintech (#20597-1-AP) | 1:1,000 (WB) |
| PDCD6IP (Alix) | - | Rabbit | Proteintech (#12422-1-AP) | 1:1,000 (WB) |
| Goat anti-Rabbit IgG HRP-linked | - | Goat | Cell Signaling (#7074) | 1:3,000 (WB) |

WB, western blotting.

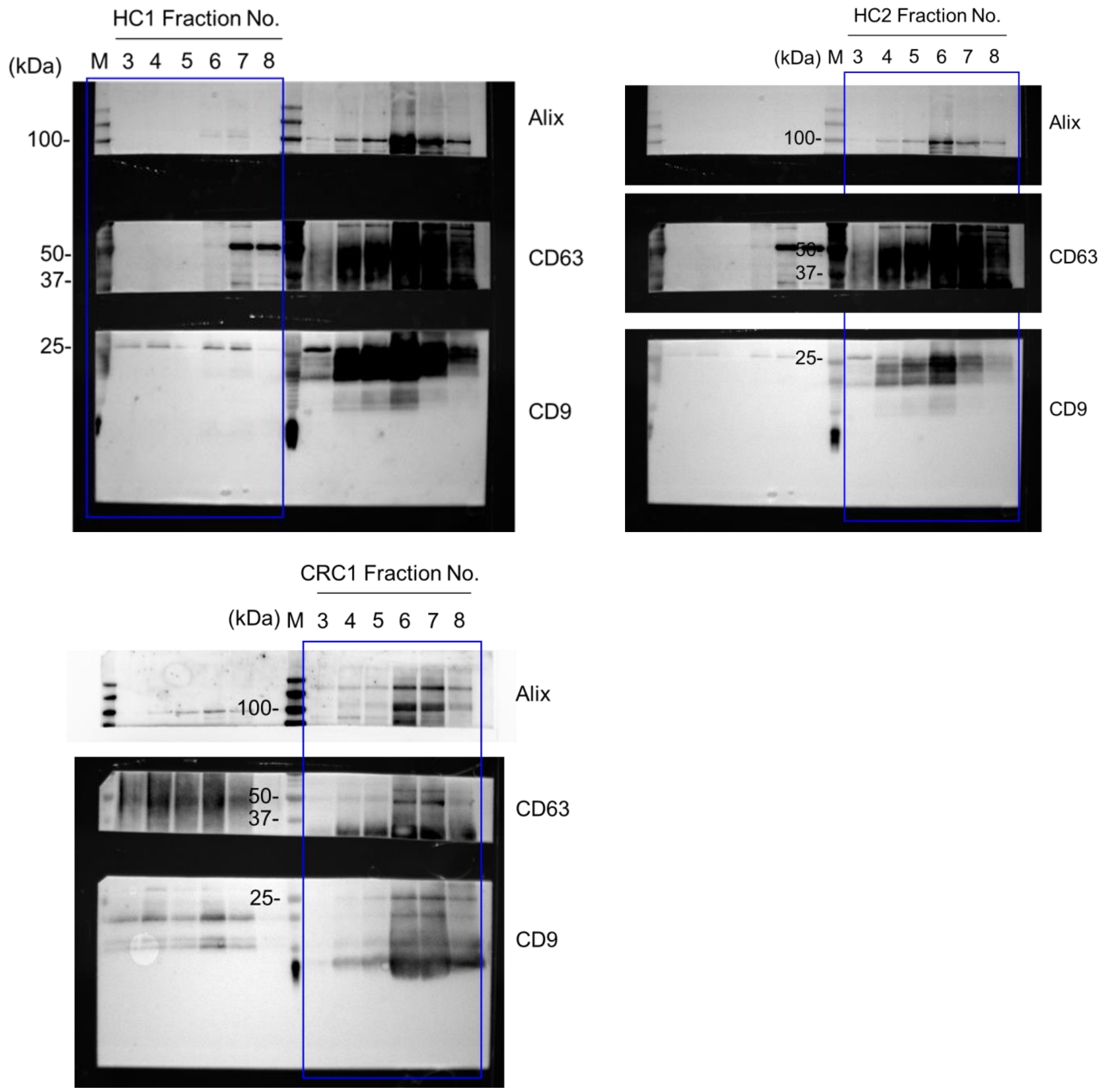


**Supplementary Figure S1. Whole images of western blotting analysis in Fig 1a.**

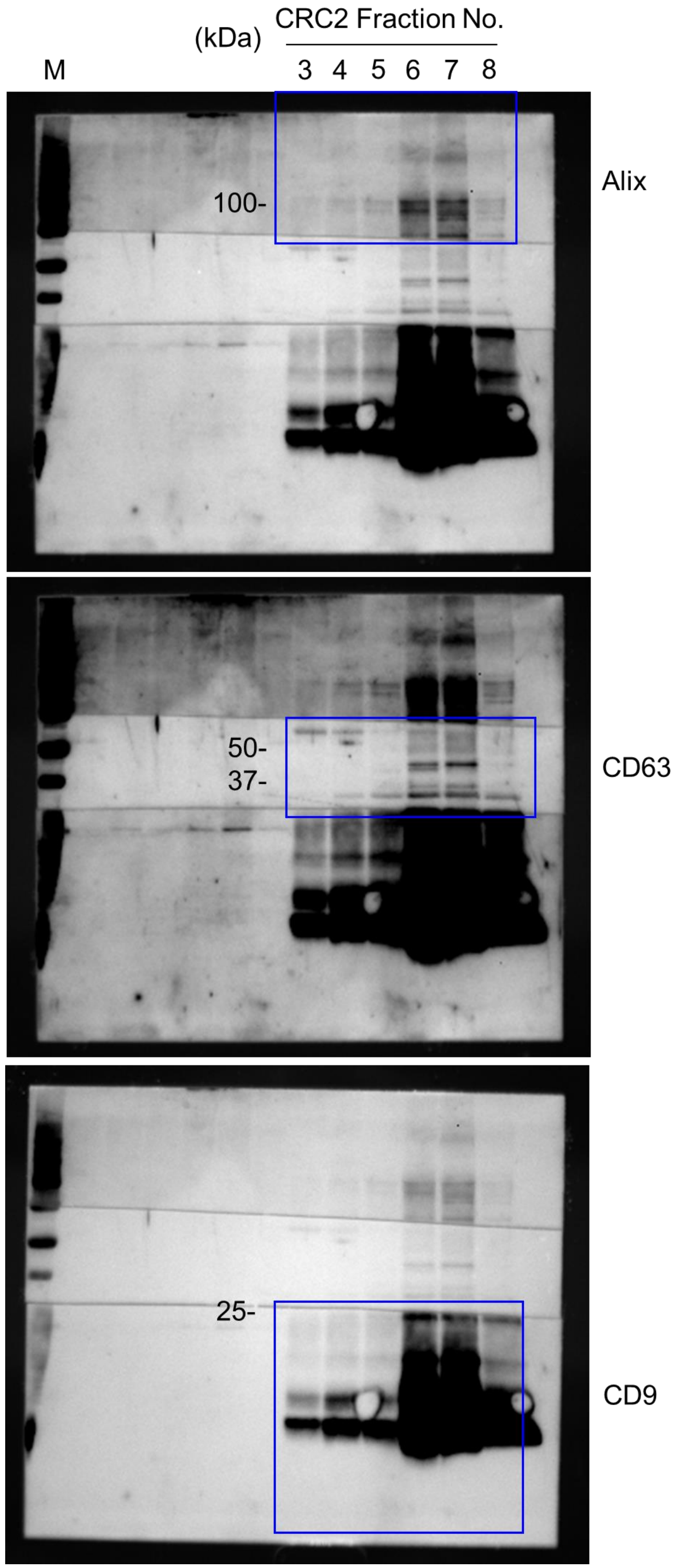


**Supplementary Figure S1. Whole images of western blotting analysis in Fig 1a.**
Alix, CD63, and CD9 levels in fEVs of HCs and patients with CRC are shown. The positions of molecular size markers (lane M) and their molecular masses in kDa on the left side. The area surrounded by the blue line was the area used in Fig. 1a.
HC; healthy control, CRC; colorectal cancer, fEV; faecal extracellular vesicle.

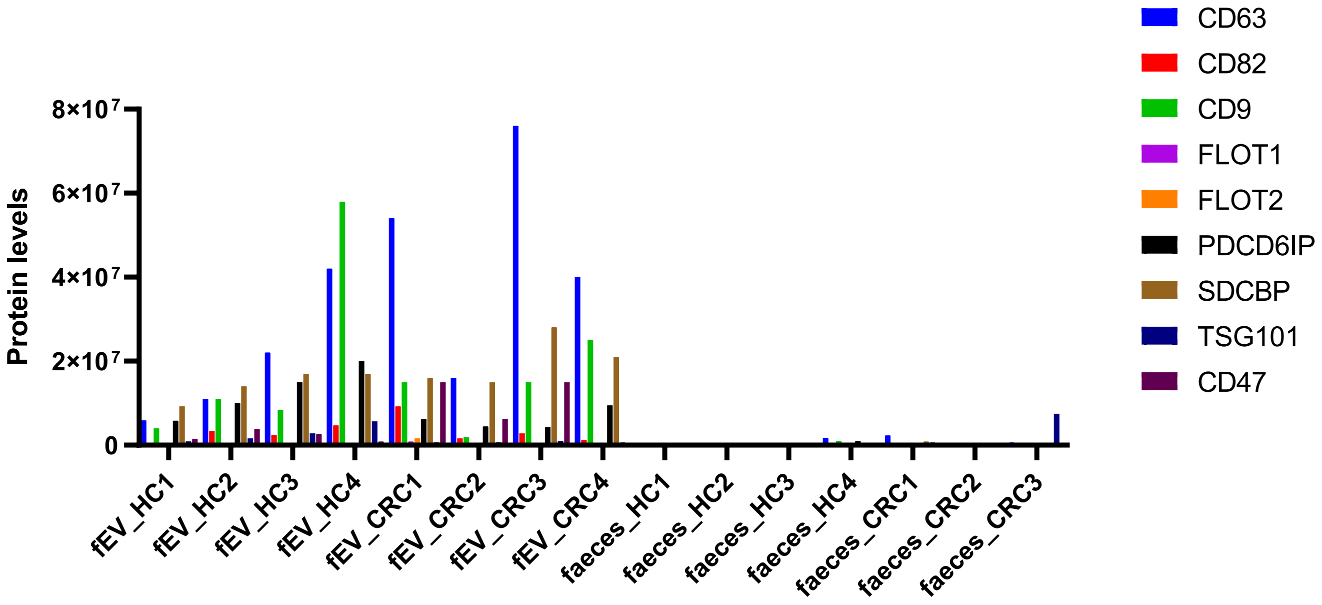


**Supplementary Figure S2. Protein levels of extracellular vesicle markers in fEVs and faeces samples.**

Graph showing quantitative values of extracellular vesicle markers in fEV and faecal suspensions obtained from HCs and patients with CRC by proteome analysis.

HC, healthy control; CRC, colorectal cancer; fEV, faecal extracellular vesicle.

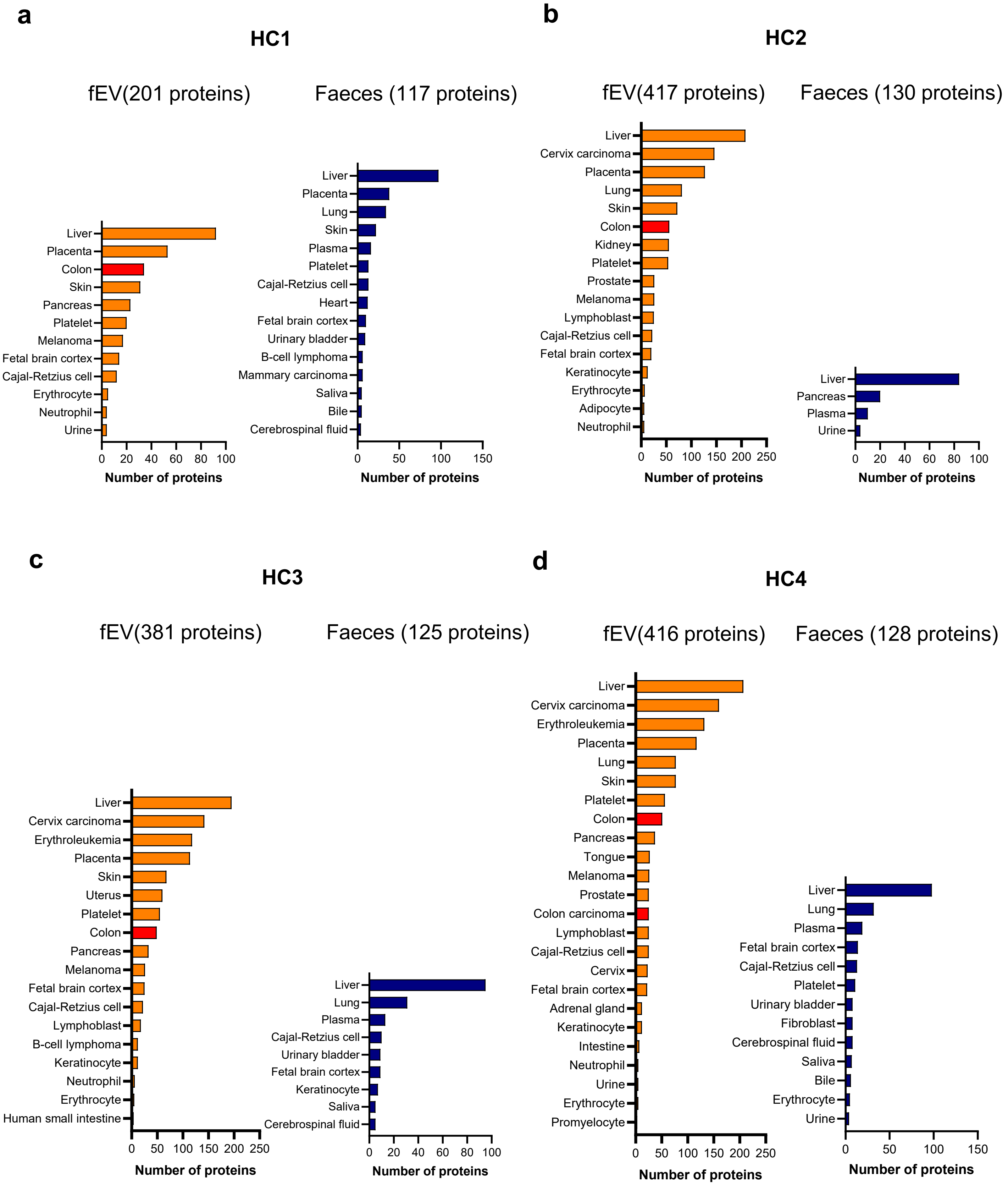


**Supplementary Figure S3. Enrichment analysis of individual HCs.**

Results of enrichment analysis in individual samples of HC. The orange bars on the left side showed the derived tissues that were enriched in the protein groups identified in fEVs only. The dark blue bars on the right side showed the derived tissues that were enriched in the protein groups identified in the faecal suspensions only. Red bars indicated colon-related tissue.

HC, healthy control; fEV, faecal extracellular vesicle.

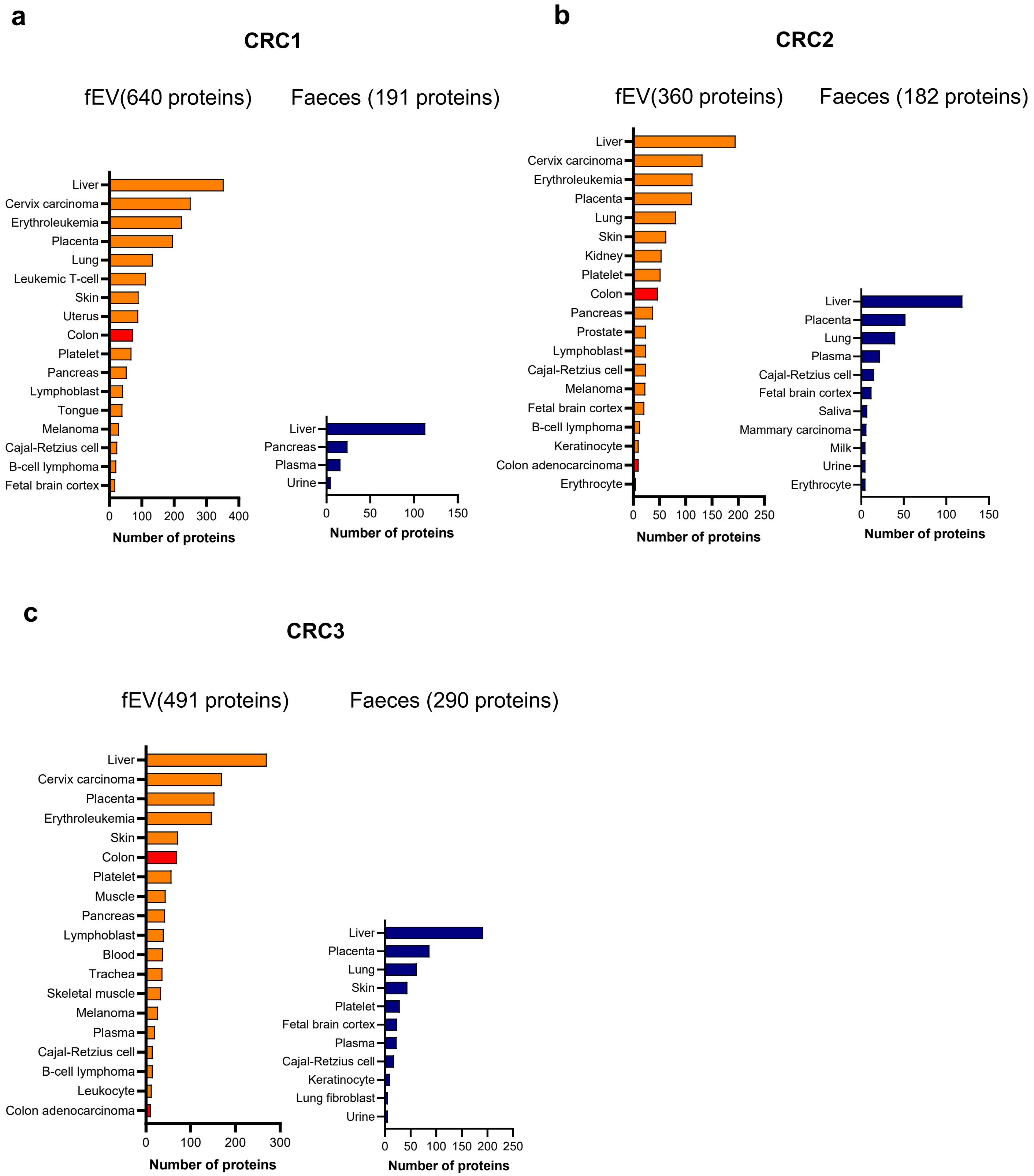


**Supplementary Figure S4. Enrichment analysis of individual patients with CRC.** Results of enrichment analysis in individual samples of patients with CRC. The orange bars on the left side showed the derived tissues that were enriched in the protein groups identified in fEVs only. The dark blue bars on the right side showed the derived tissues that were enriched in the protein groups identified in the faecal suspension only. Red bars indicated colon-related tissue.

CRC, colorectal cancer; fEV, faecal extracellular vesicle.

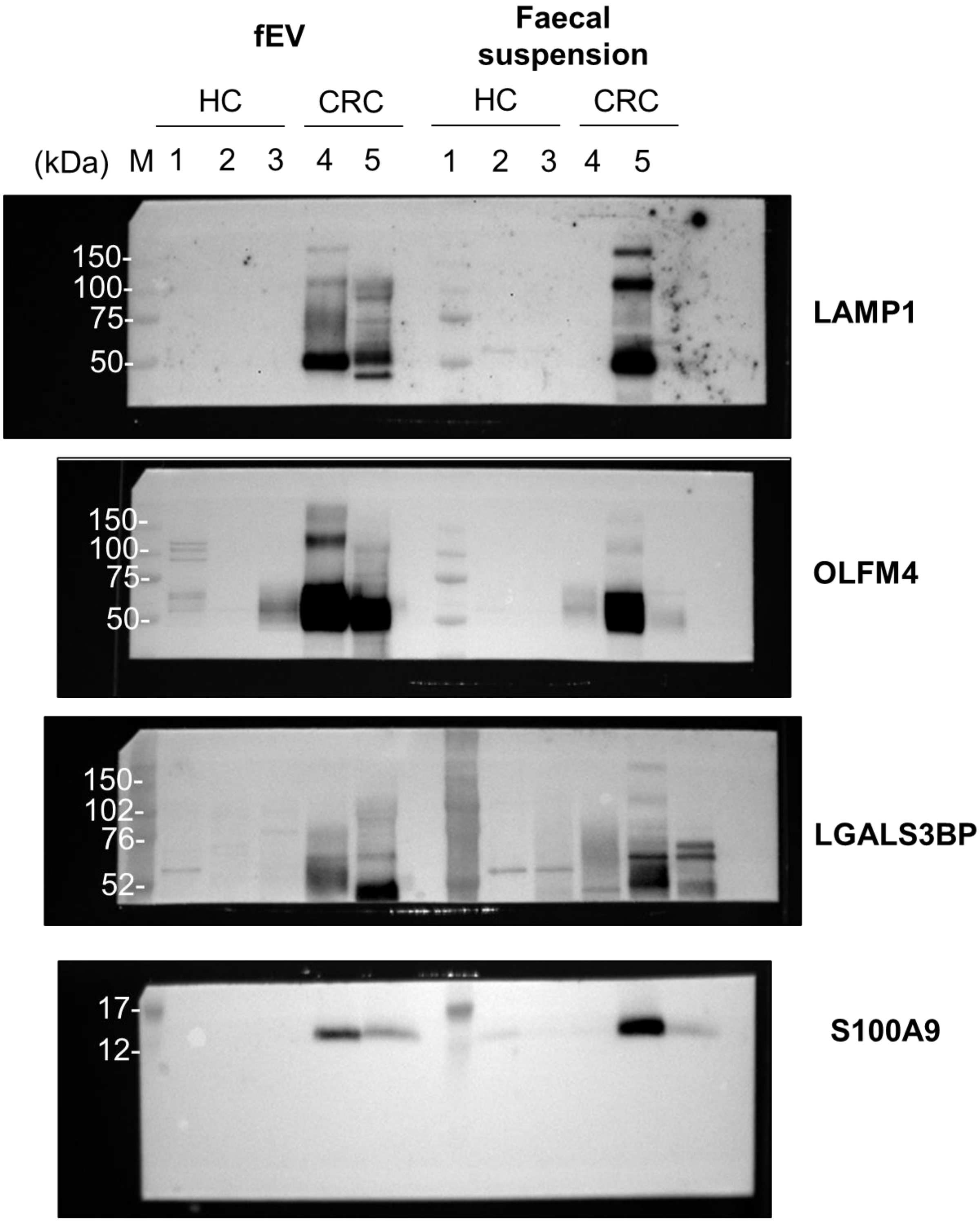


**Supplementary Figure S5. Whole images of western blotting analysis in Fig 3b.**
LAMP1, OLFM4,LGALS3BP, and S100A9 levels in fEVs or faecal suspensions of HCs and patients with CRC are shown. The positions of molecular size markers (lane M) and their molecular masses in kDa on the left side. All images were a half gel image of a high molecular or low molecular weight sides.
HC; healthy control, CRC; colorectal cancer, fEV; faecal extracellular vesicle.

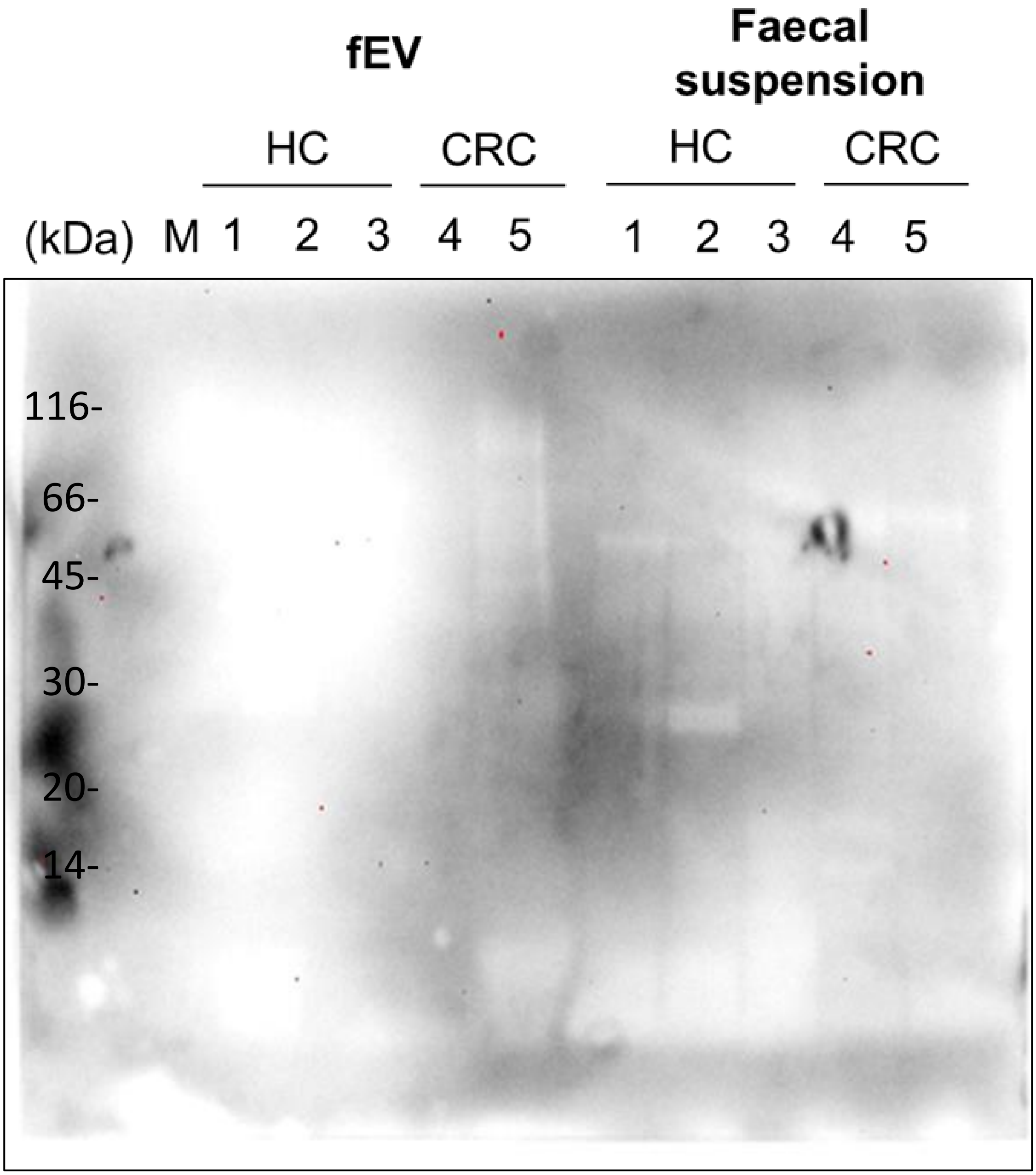


**Supplementary Figure S6. Primary antibody omission plot for western blotting analysis in Fig 3b.**
HC; healthy control, CRC; colorectal cancer, fEV; faecal extracellular vesicle.

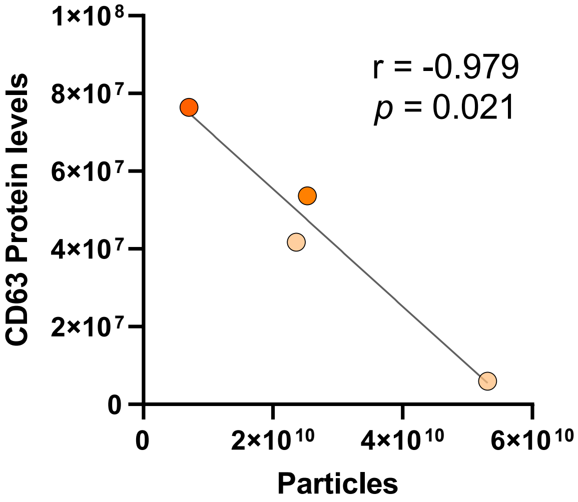


**Supplementary Figure S7. Correlation between the quantification values of CD63 and the EV particles.**

Correlation between CD63 quantification values obtained from proteome analysis and the number of EV particles in the sample used for proteome analysis. The samples were HC1 and HC2, CRC1, and CRC2 as shown in Fig. 1. Orange plots indicated CRCs.

HC; healthy control, CRC; colorectal cancer.

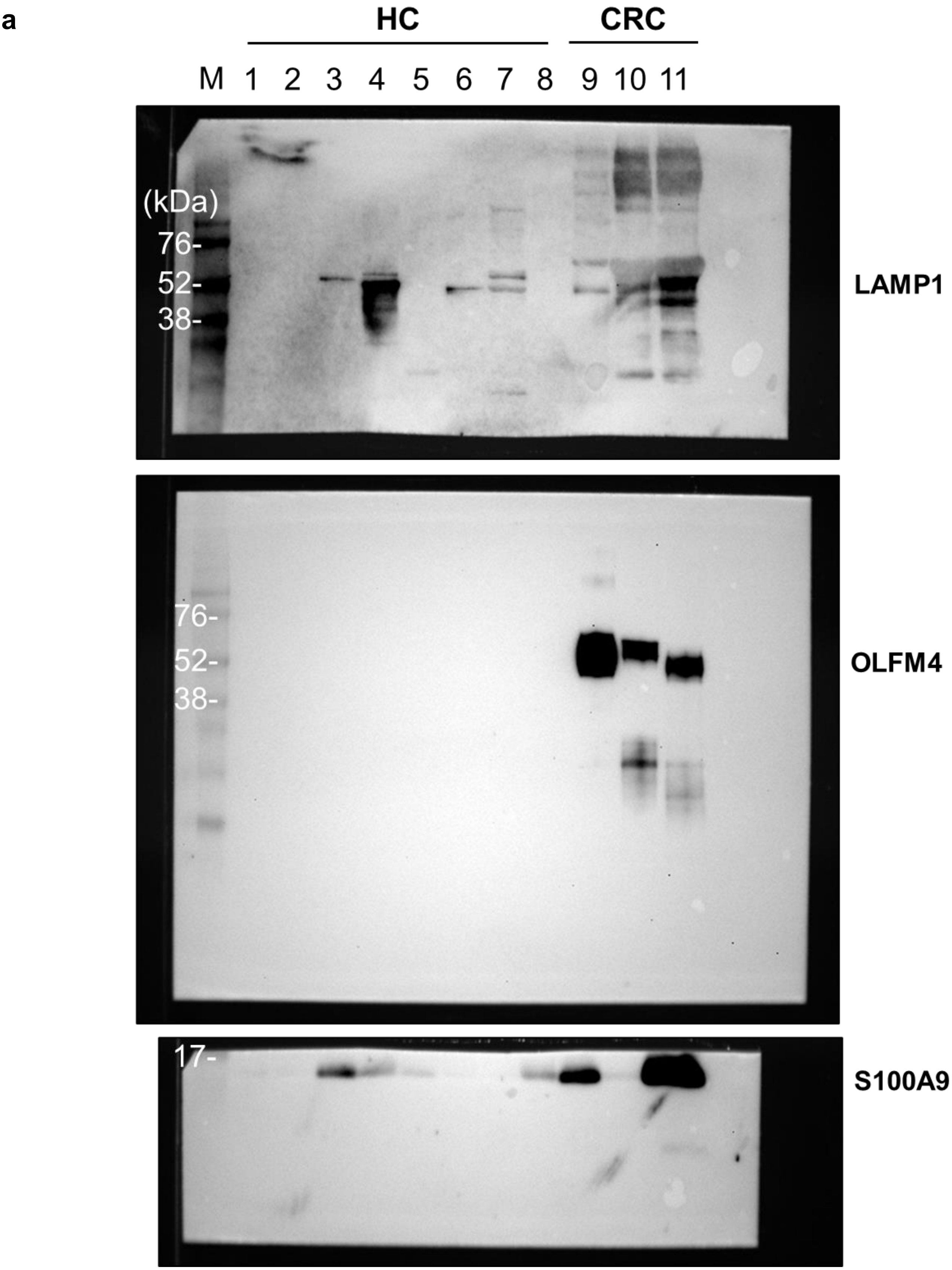


**Supplementary Figure S8. Whole images of western blotting analysis in Fig 4b.**

b

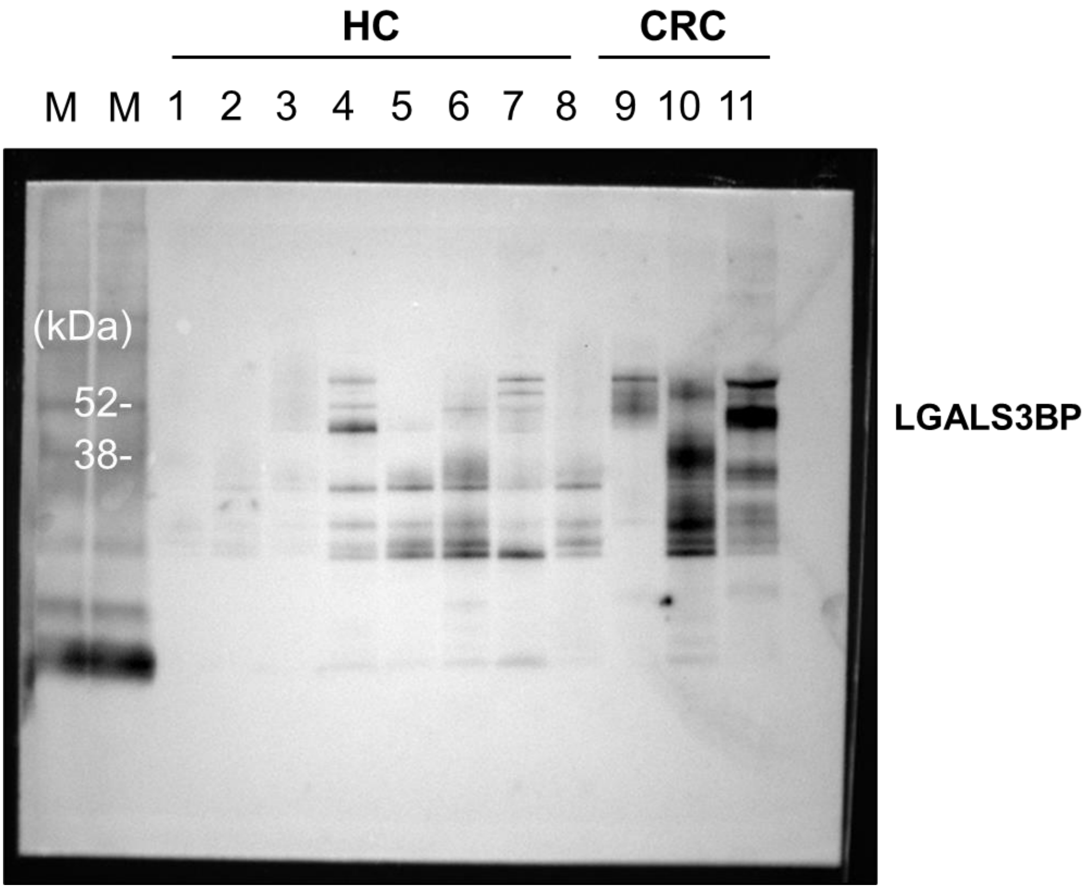


**Supplementary Figure S8. Whole images of western blotting analysis in Fig 4b.**
LAMP1, OLFM4,LGALS3BP, and S100A9 levels in fEVs of HCs and patients with CRC were shown. The positions of molecular size markers (lane M) and their molecular masses in kDa on the left side. LAMP1 and S100A9 were a half gel image of a high or low molecular weight side. OLFM4 and LAMP1 were shown a total gel image.
HC; healthy control, CRC; colorectal cancer, fEV; faecal extracellular vesicle.
